# Pharmacological Management of Secondary Hyperparathyroidism in Dialysis Patients: A Narrative Review of Calcimimetics, Vitamin D Analogs, and Phosphate Binders

**DOI:** 10.7759/cureus.111377

**Published:** 2026-06-23

**Authors:** Hanin Alwashahi, Dhanalekshmi Unnikrishnan Meenakshi

**Affiliations:** 1 College of Pharmacy, National University of Science and Technology, Muscat, OMN; 2 Department of Pharmacy, Ministry of Health, North Al Batinah, OMN

**Keywords:** calcimimetics, chronic kidney disease, dialysis patients, parathyroid hormone control, pharmacological management, phosphate binders, secondary hyperparathyroidism, vitamin d analogs

## Abstract

Secondary hyperparathyroidism (sHPT) is a prevalent complication of end-stage renal disease, driven by phosphate retention, vitamin D deficiency, hypocalcemia, and calcium-sensing receptor dysregulation. Despite international guidelines, optimal biochemical control remains challenging in real-world practice. This narrative review synthesizes current evidence on the pharmacological management of sHPT in dialysis patients, with attention to the underrepresented Middle Eastern and Gulf Cooperation Council (GCC) context. A narrative review was conducted using PubMed and Scopus, identifying original clinical studies, randomized controlled trials, and observational cohorts examining calcimimetics, vitamin D analogs, and phosphate binders in dialysis patients with sHPT. In total, 22 studies were included. Calcimimetics, particularly cinacalcet, reduce parathyroid hormone (PTH) by approximately 43% in hemodialysis patients without raising serum calcium, though hypocalcemia and gastrointestinal adverse effects limit tolerability. Intravenous etelcalcetide offers superior PTH suppression with improved gastrointestinal tolerability and eliminates oral adherence challenges through supervised dialysis-circuit administration. A large trial demonstrated no mortality benefit despite sustained PTH lowering. Vitamin D analogs suppress PTH gene transcription but carry a narrow therapeutic window with risks of hypercalcemia and hyperphosphatemia. Phosphate binders address the primary upstream driver of sHPT; non-calcium-based agents such as sevelamer offer cardiovascular advantages but impose a pill burden contributing to nonadherence rates of 22-74%. Novel agents including tenapanor offer complementary phosphate-lowering mechanisms with substantially reduced pill burden. Combination therapy allows dose reduction of individual agents while optimizing biochemical outcomes. Evidence from Middle Eastern and GCC dialysis populations is virtually absent. Pharmacological management of sHPT requires individualized, guideline-informed combination therapy. A critical evidence gap exists in the GCC region, where diabetic nephropathy dominance, endemic vitamin D deficiency, and lower calcimimetic utilization may limit generalizability of existing trial data. Locally conducted research is urgently needed.

## Introduction and background

Chronic kidney disease (CKD) is a progressive disorder characterized by the gradual loss of renal capacity to filter waste products and regulate fluid and electrolyte balance. When kidney function deteriorates sufficiently, toxic metabolites accumulate, and severe systemic complications follow. CKD now affects more than 10% of the global population and represents a major source of morbidity and mortality worldwide [1]. The increasing prevalence of CKD is closely linked to the growing burden of diabetes mellitus, hypertension, obesity, and an aging population [2].

When CKD progresses to end-stage renal disease (ESRD), renal replacement therapy, either dialysis or kidney transplantation, becomes necessary for survival. Dialysis corrects solute accumulation but cannot fully restore the kidney’s endocrine functions, and patients on long-term dialysis consequently develop persistent metabolic disturbances requiring continuous management, of which secondary hyperparathyroidism (sHPT) is among the most clinically consequential [3].

sHPT develops through interconnected metabolic disruptions in CKD: phosphate retention, reduced synthesis of active vitamin D (calcitriol), and hypocalcemia collectively drive progressive parathyroid gland hyperplasia and sustained overproduction of parathyroid hormone (PTH) [4]. This process is a central feature of CKD-mineral and bone disorder (CKD-MBD) [5]. Among hemodialysis patients in Gulf Cooperation Council (GCC) countries, approximately 20% have severely elevated PTH levels exceeding 700 pg/mL, with median values ranging from 259 to 437 pg/mL across participating centers [6]. Both very low and very high PTH levels have been independently associated with increased mortality [7], underscoring the clinical importance of achieving and maintaining biochemical targets.

Uncontrolled sHPT carries serious skeletal and cardiovascular consequences. At the bone level, persistently elevated PTH drives excessive osteoclast activity, producing osteitis fibrosa cystica, a high-turnover bone disease associated with pain, deformity, and markedly increased fracture risk [8]. Conversely, oversuppression of PTH leads to adynamic bone disease, a low-turnover state that impairs normal remodeling and predisposes to fracture [8]. Therefore, managing sHPT requires avoiding both extremes. Even moderate CKD substantially elevates fracture risk, and dialysis patients experience fracture rates that far exceed those of the general population; fractures in this setting carry a 3.7-fold increase in mortality risk [9]. Cardiovascular disease accounts for approximately 40-50% of all-cause mortality in dialysis patients, and dysregulated mineral metabolism is a major contributing mechanism [7,10]. Elevated phosphate and calcium-phosphate product drive vascular smooth muscle cells toward an osteogenic phenotype, resulting in medial arterial calcification and arterial stiffness [11]. Observational data further indicate that elevated PTH levels are associated with increased risk of hospitalization and mortality across dialysis populations [6,7].

Persistently elevated PTH despite treatment has been documented across major dialysis registries globally, reflecting the limitations of available pharmacological strategies [6,7]. Mineral metabolism disturbances are more severe and harder to correct at the dialysis stage than in earlier CKD [3], and, collectively, international data indicate that sHPT remains common and inadequately controlled across many dialysis facilities, pointing to the need for systematic surveillance and continuous treatment optimization [6].

The GCC region carries a high burden of CKD risk factors; type 2 diabetes, hypertension, and obesity are highly prevalent, and their rates have risen sharply over recent decades [6]. Cinacalcet utilization in GCC countries is substantially lower than in European and North American centers, and multinational comparisons have identified significant regional variation in phosphate binder use, vitamin D prescribing, and overall CKD-MBD stewardship [6]. Vitamin D deficiency adds another layer of complexity in this population. Despite abundant sunlight, vitamin D insufficiency is prevalent across Arab and Gulf populations due to limited sun exposure, indoor lifestyles, and cultural clothing practices [12]. Baseline vitamin D deficiency reduces calcitriol synthesis, blunts PTH suppression, and may worsen the severity of sHPT before dialysis-specific pharmacotherapy is even initiated [4,12]. Regional dietary habits and genetic factors further shape the clinical picture, meaning that evidence generated in Western or Asian trial populations cannot be assumed to apply directly to Gulf patients.

In Oman, diabetes mellitus affects approximately 15.7% of adults [13], and diabetic nephropathy is the leading cause of ESRD, accounting for 46% of all incident dialysis cases in the national RRT registry [14]. The dialysis population has grown substantially over the past two decades alongside improvements in life expectancy and expansion of dialysis infrastructure across governorates. With this growth comes an increasing burden of CKD-MBD complications, including sHPT, that remains inadequately characterized in the Omani clinical literature. No published peer-reviewed studies have specifically examined the pharmacological management of sHPT in Omani dialysis patients. This is a clinically meaningful gap. Omani patients may differ from Western or Asian trial populations in ways that are directly relevant to treatment response: dietary phosphate exposure shaped by regional food patterns, genetic influences on drug metabolism, distinct comorbidity profiles, and healthcare access patterns that affect both treatment initiation and adherence. Clinical pharmacists and nephrologists in Oman currently make treatment decisions based entirely on evidence generated elsewhere, an assumption that has never been directly tested in this population and that deserves examination.

Three main pharmacological classes are used to manage sHPT: phosphate binders, active vitamin D analogs, and calcimimetics, used alone or in combination guided by laboratory findings and clinical status [5]. Phosphate binders reduce intestinal phosphate absorption to address a primary upstream driver of sHPT. Calcium-based agents such as calcium carbonate and calcium acetate are effective and widely available but carry risks of calcium loading and vascular calcification with prolonged use [15,16]. Non-calcium-based agents, including sevelamer, avoid these risks and may confer cardiovascular advantages through attenuation of arterial calcification [15], though at higher cost and pill burden. Active vitamin D analogs, i.e., calcitriol and alfacalcidol, suppress PTH gene transcription and enhance calcium absorption; however, their therapeutic window is narrow, and the risks of hypercalcemia and hyperphosphatemia require careful monitoring [17]. Calcimimetics such as cinacalcet increase calcium-sensing receptor sensitivity in parathyroid tissue, effectively lowering PTH release without raising serum calcium [18]. They are frequently used alongside vitamin D analogs to achieve broader biochemical control than either class alone. Current Kidney Disease: Improving Global Outcomes (KDIGO) guidance emphasizes individualized CKD-MBD management informed by trends in PTH, calcium, and phosphate rather than rigid numerical targets [5].

Despite meaningful progress in sHPT pharmacotherapy, critical evidence gaps remain. Most clinical trials have been powered to detect biochemical endpoints rather than hard clinical outcomes such as fractures, hospitalizations, or mortality, a limitation illustrated by the large randomized trial of cinacalcet that failed to demonstrate mortality reduction despite clear PTH-lowering efficacy [19]. Evidence from Middle Eastern populations is virtually absent, leaving a regional evidence base that clinicians in GCC countries cannot rely on with confidence [6]. Combination therapy strategies lack practical guidance on sequencing and dose adjustment in real-world settings. Phosphate binder nonadherence, driven by high pill counts and gastrointestinal intolerance, remains a significant and underaddressed challenge, with reported nonadherence rates ranging from 22% to 74% across dialysis populations [20]. Furthermore, the cost-effectiveness of available agents has not been evaluated using GCC-specific drug pricing and utilization data.

This narrative review aims to synthesize current evidence on the pharmacological management of sHPT in dialysis patients; critically evaluate the comparative effectiveness and limitations of calcimimetics, vitamin D analogs, and phosphate binders; assess guideline implementation gaps in real-world clinical practice; and highlight the specific evidence gaps relevant to Middle Eastern and GCC dialysis populations where locally generated data remain absent.

## Review

Methodology

A narrative literature search was conducted using PubMed and Scopus databases, covering publications from 1996 to 2024. The following search terms were used individually and in combination: secondary hyperparathyroidism, dialysis, hemodialysis, end-stage renal disease, calcimimetics, cinacalcet, etelcalcetide, vitamin D analogues, paricalcitol, calcitriol, alfacalcidol, phosphate binders, sevelamer, lanthanum carbonate, calcium carbonate, CKD-MBD, parathyroid hormone, FGF-23, vascular calcification, Gulf Cooperation Council, and Oman. Searches were limited to English-language publications. Studies were selected based on clinical relevance, methodological quality, and applicability to dialysis populations. Priority was given to randomized controlled trials, large observational cohort studies, registry-based analyses, and current clinical practice guidelines, particularly KDIGO 2017. Narrative reviews and editorials were excluded unless they provided essential contextual information unavailable in primary sources. Study quality was assessed narratively, with explicit acknowledgement of study design limitations where relevant, including the distinction between randomized and observational evidence and the risk of confounding in non-randomized studies. The authors acknowledge that as a narrative rather than systematic review, this work does not follow Preferred Reporting Items for Systematic Reviews and Meta-Analyses (PRISMA) guidelines and is subject to potential selection bias in study inclusion. Findings are therefore intended to provide a clinically oriented synthesis of available evidence rather than a definitive comparative effectiveness analysis.

Pathophysiology of secondary hyperparathyroidism

sHPT develops through multiple interconnected mechanisms in CKD. The primary drivers include phosphate retention, elevated fibroblast growth factor-23 (FGF-23), and vitamin D deficiency [4,21]. As glomerular filtration rate (GFR) declines, the kidneys progressively lose their capacity to excrete dietary phosphate. The resulting hyperphosphatemia directly stimulates PTH gene transcription and secretion in the parathyroid glands, an effect that is independent of calcium levels and operates even at phosphate concentrations within the normal range [4]. Simultaneously, impaired renal 1-alpha hydroxylation of 25-hydroxyvitamin D reduces calcitriol synthesis, diminishing its suppressive effect on PTH gene transcription and reducing intestinal calcium absorption, resulting in hypocalcemia that further drives parathyroid activity. The calcium-sensing receptor (CaSR) on parathyroid cells, which normally suppresses PTH release in response to rising calcium, becomes downregulated in the setting of persistent uremic stimulation, reducing the parathyroid gland’s sensitivity to calcium and further amplifying PTH secretion [4,18]. FGF-23 plays an important early role in this cascade. Produced by osteocytes in response to rising phosphate, FGF-23 acts on the kidney to promote phosphate excretion and suppress calcitriol production [21]. In early CKD, elevated FGF-23 can maintain phosphate homeostasis; however, as nephron loss advances, this compensatory mechanism fails, and hyperphosphatemia ensues. Notably, FGF-23 rises before PTH and phosphate become overtly abnormal, making it an early biomarker of CKD-MBD [21]. Collectively, these disturbances lead to progressive parathyroid gland hyperplasia. Initially, the hyperplasia is diffuse and potentially reversible with medical therapy; however, with sustained stimulation, nodular transformation occurs within the enlarged glands, rendering the tissue autonomous and resistant to pharmacological suppression [8]. This biological progression explains why sHPT that has been present for many years responds poorly to medical management and may ultimately require parathyroidectomy [8]. This biological progression underscores the clinical importance of early intervention before irreversible glandular changes are established.

Treatment guidelines

The KDIGO 2017 clinical practice guidelines provide the principal evidence-based framework for CKD-MBD management [5]. For dialysis patients, the guidelines recommend maintaining intact PTH (iPTH) at two to nine times the upper limit of normal for the assay used, reflecting recognition that both oversuppression and undertreatment carry independent mortality risks [5,7]. Crucially, KDIGO 2017 moved away from fixed numerical PTH targets, emphasizing instead the monitoring of trends over time and the integration of multiple biochemical parameters, i.e., calcium, phosphate, alkaline phosphatase, and PTH, rather than any single value in isolation [5]. This represents a significant evolution from earlier guideline iterations, which specified narrower target ranges that were associated with overtreatment and iatrogenic adynamic bone disease in some patients [8]. KDIGO endorses the use of calcimimetics, phosphate binders, and vitamin D analogs, either alone or in combination, while explicitly acknowledging that no specific pharmacological intervention has been demonstrated to reduce mortality in randomized controlled trials [5]. For patients with progressive or severe hyperphosphatemia, phosphate binders are recommended as first-line intervention. Active vitamin D analogs or calcimimetics are indicated for persistent PTH elevation after phosphate control is addressed, with the choice between agents guided by individual calcium levels, tolerability, and cost considerations [5]. The guidelines do not mandate a specific sequencing of therapies, instead endorsing individualized clinical decision-making that accounts for patient-specific biochemical profiles, dialysis modality, comorbidities, and tolerability of prior treatments [5].

Calcimimetics

Calcimimetics act by allosterically increasing the sensitivity of CaSRs on parathyroid gland chief cells, shifting the receptor response curve so that existing ambient calcium concentrations produce greater PTH suppression [18]. This mechanism is distinct from vitamin D analogs: calcimimetics lower PTH without raising serum calcium or phosphate, making them particularly suitable for patients with hypercalcemia or hyperphosphatemia in whom vitamin D analogs are contraindicated or poorly tolerated [18]. Cinacalcet hydrochloride is the most extensively studied oral calcimimetic. In a randomized controlled trial of 741 hemodialysis patients with uncontrolled sHPT, cinacalcet reduced median PTH by approximately 43% from baseline, with 43% of patients achieving PTH ≤250 pg/mL compared with 5% in the placebo group [18]. Reductions in serum phosphate and calcium-phosphate product were also observed, effects that carry independent cardiovascular significance given the established association between hyperphosphatemia and vascular calcification [10,18]. The most frequently reported adverse effects are hypocalcemia and gastrointestinal symptoms, including nausea, vomiting, and diarrhea, which can limit tolerability and lead to dose reduction or discontinuation [18]. Serum calcium monitoring is essential during cinacalcet dose titration, and co-administration of calcium supplements or active vitamin D may be required to manage symptomatic hypocalcemia.

A large randomized controlled trial of cinacalcet (EVOLVE trial) involving over 3,800 hemodialysis patients failed to demonstrate a statistically significant reduction in cardiovascular events or mortality despite clear PTH-lowering efficacy [19]. The intention-to-treat analysis yielded a non-significant hazard ratio of 0.93 for the primary composite cardiovascular endpoint, while an adjusted analysis suggested a possible 12% relative risk reduction (hazard ratio = 0.88), though this post-hoc finding requires cautious interpretation. The trial was substantially complicated by a 58% rate of study drug discontinuation in the cinacalcet arm and significant placebo crossover, which severely limited interpretability [19]. This finding serves as an important reminder that biochemical improvement does not automatically translate into improved clinical outcomes, and that the cardiovascular pathophysiology of dialysis patients is multifactorial in nature [19]. Regarding newer calcimimetic agents, etelcalcetide is an intravenous second-generation calcimimetic that represents an important advance over oral cinacalcet in several clinically meaningful respects. Administered intravenously three times weekly via the dialysis circuit at the end of each hemodialysis session, etelcalcetide eliminates the daily oral adherence challenges associated with cinacalcet, as administration is directly supervised by dialysis staff. Head-to-head randomized controlled trial evidence has demonstrated that etelcalcetide achieves superior PTH suppression compared to cinacalcet, with a statistically significantly higher proportion of patients achieving greater than 30% PTH reduction from baseline. Gastrointestinal adverse effects are significantly less frequent with etelcalcetide compared to cinacalcet, representing a meaningful clinical advantage for patients who have previously experienced cinacalcet intolerance. However, etelcalcetide is associated with a higher incidence of hypocalcemia compared to cinacalcet in direct comparative trials, requiring careful calcium monitoring, particularly in patients with borderline or low baseline calcium levels. Access to etelcalcetide in GCC healthcare systems remains limited due to higher acquisition costs and restricted formulary inclusion. Evocalcet is a next-generation oral calcimimetic with a modified chemical structure designed to reduce gastrointestinal side effects compared to cinacalcet. Clinical trial evidence from hemodialysis patients demonstrated non-inferiority to cinacalcet in PTH reduction with a significantly lower incidence of gastrointestinal adverse events, suggesting an improved tolerability profile for patients requiring oral calcimimetic therapy. Evocalcet is currently approved in Japan but is not yet widely available in GCC healthcare systems. In the GCC context, where calcimimetic utilization is substantially lower than in Western centers due to cost barriers, formulary restrictions, and tolerability concerns, the availability of intravenous etelcalcetide and improved oral agents represents clinically important therapeutic advances that deserve specific consideration in regional formulary decisions.

Vitamin D analogs

Vitamin D analogs suppress PTH synthesis by binding to vitamin D receptors (VDRs) in parathyroid cell nuclei, directly inhibiting PTH gene transcription and reducing parathyroid cell proliferation [17]. They simultaneously enhance intestinal calcium absorption, raising serum calcium and providing additional indirect PTH suppression through the CaSR pathway. This dual mechanism makes vitamin D analogs effective PTH-lowering agents, particularly in patients with mild-to-moderate sHPT and low or normal serum calcium [17]. Calcitriol (1,25-dihydroxyvitamin D3) is the biologically active form and the original agent used in CKD. Selective vitamin D receptor activators (SVDRAs), including paricalcitol and doxercalciferol, were subsequently developed to achieve PTH suppression with reduced calcemic and phosphatemic effects [17]. In a randomized controlled trial comparing paricalcitol with calcitriol in hemodialysis patients, both agents produced significant PTH reductions, but paricalcitol was associated with fewer episodes of hypercalcemia [17]. Observational data from a large cohort of hemodialysis patients suggested that paricalcitol therapy was associated with improved survival compared with calcitriol, with an approximate 16% relative risk reduction in all-cause mortality [22]. While the observational design precludes causal conclusions, these findings have influenced prescribing patterns and contributed to the wider adoption of SVDRAs in dialysis units globally.

The evolution of vitamin D therapy in CKD has been marked by historical shifts from liberal to more restrained use, driven by accumulating evidence of risks associated with hypercalcemia, hyperphosphatemia, and vascular calcification [7,22]. The principal limitation of vitamin D therapy remains its narrow therapeutic window: achieving adequate PTH suppression while avoiding calcium and phosphate elevation requires careful dose titration and regular laboratory monitoring [17]. In patients with baseline hypercalcemia or severe hyperphosphatemia, vitamin D analogs may need to be withheld or used at reduced doses, limiting their applicability across the full spectrum of dialysis patients [5].

Phosphate binders

Phosphate binders are a cornerstone of sHPT management, directly targeting one of the primary upstream pathophysiological drivers by reducing intestinal absorption of dietary phosphate, thereby attenuating the phosphate stimulus on PTH secretion [4]. The KDIGO guidelines recommend phosphate binders as first-line pharmacological therapy for hyperphosphatemia in dialysis patients, with a target serum phosphate of 1.13-1.78 mmol/L (3.5-5.5 mg/dL), while emphasizing that dietary phosphate restriction remains an essential non-pharmacological adjunct [5].

Available phosphate binders fall into two main categories: calcium-based agents (calcium carbonate, calcium acetate) and non-calcium-based agents (sevelamer, lanthanum carbonate). Calcium-based binders are widely accessible and effective but carry risks of hypercalcemia and progressive vascular calcification, particularly when used concurrently with active vitamin D therapy [16]. Calcium acetate binds phosphate more efficiently per gram of elemental calcium than calcium carbonate, resulting in lower net calcium absorption for equivalent phosphate control, which may reduce the risk of hypercalcemia with long-term use [16]. In a randomized controlled trial enrolling patients new to hemodialysis and followed over 18 months, calcium-based binders were associated with significantly greater progression of coronary artery calcification compared with sevelamer, highlighting the cardiovascular risk of sustained calcium loading in the dialysis population [16]. Sevelamer hydrochloride and sevelamer carbonate are non-absorbed polymeric resins that lower serum phosphate without contributing to calcium load. A randomized controlled trial found that sevelamer attenuated the progression of coronary and aortic calcification compared with calcium carbonate despite equivalent phosphate-lowering efficacy, with additional benefits potentially related to its lipid-lowering and anti-inflammatory properties [15]. Lanthanum carbonate represents another non-calcium alternative that binds phosphate across a wide gastrointestinal pH range; however, concerns regarding potential tissue accumulation with prolonged use have led to more cautious prescribing in some centers. In resource-limited GCC healthcare settings, non-calcium binders face a significant cost barrier, with sevelamer substantially more expensive than calcium-based alternatives and formulary access often restricted to patients demonstrating intolerance or inadequate control with calcium agents [6].

The practical burden of phosphate binder therapy is substantial and constitutes one of the most important modifiable barriers to effective sHPT management. Dialysis patients typically require six to twelve binder tablets daily, taken with every meal and snack, in addition to their full medication regimen [20]. Gastrointestinal side effects, including nausea, constipation, diarrhea, and bloating, are common across all binder classes and vary by agent and formulation [20]. These factors collectively drive non-adherence rates estimated at 22% to 74% across dialysis populations, making phosphate binder non-adherence the single most prevalent modifiable contributor to persistent hyperphosphatemia [20]. Persistent hyperphosphatemia resulting from non-adherence not only maintains the primary phosphate stimulus for PTH secretion but can also counteract the biochemical effects of concurrently administered calcimimetics and vitamin D analogs, fundamentally undermining the overall pharmacological strategy for sHPT management [5]. A novel approach to phosphate control that addresses the pill burden limitation of conventional binders is tenapanor, a first-in-class sodium-hydrogen exchanger isoform 3 (NHE3) inhibitor that represents a paradigm shift in the management of hyperphosphatemia. Unlike conventional phosphate binders that chemically bind dietary phosphate within the intestinal lumen, tenapanor blocks the paracellular transport pathway through which phosphate is passively absorbed across intestinal epithelial cells by reducing intestinal tight junction permeability to phosphate. This fundamentally different and complementary mechanism does not depend on luminal binding and does not require administration with every meal. The BLOCK randomized controlled trial demonstrated that tenapanor significantly reduced serum phosphate compared to placebo in hemodialysis patients, with a mean reduction of approximately 1.4 mg/dL from baseline. The subsequent AMPLIFY trial demonstrated that adding tenapanor to existing binder therapy produced additional statistically significant phosphate reductions beyond those achievable with binders alone, supporting its use as a complementary agent in patients with inadequately controlled hyperphosphatemia. A key clinical advantage of tenapanor is its substantially reduced pill burden, administered as twice-daily dosing independent of meals compared to the six to twelve tablets daily required with conventional binders, which has significant potential to improve real-world adherence, particularly in Middle Eastern and GCC dialysis populations where pill burden is a documented barrier to effective sHPT management. Tenapanor is currently approved by the US FDA but is not yet widely available in GCC healthcare systems, and its higher acquisition cost relative to conventional binders represents a significant barrier to formulary inclusion in cost-sensitive regional healthcare settings.

Combination therapy

Combination approaches using agents from multiple pharmacological classes are increasingly used in clinical practice to optimize biochemical control while limiting the toxicity associated with high doses of any single agent. The rationale rests on the complementary mechanisms of the three drug classes: phosphate binders reduce the phosphate stimulus driving PTH secretion; vitamin D analogs directly suppress PTH gene transcription; and calcimimetics increase parathyroid CaSR sensitivity [5]. Using these mechanisms together allows lower doses of each individual agent, potentially reducing dose-dependent adverse effects such as hypercalcemia from vitamin D analogs or gastrointestinal intolerance from cinacalcet. In practice, the most common combination is a phosphate binder with either a vitamin D analog or a calcimimetic, with the choice between the latter two guided by individual calcium levels: vitamin D analogs are preferred when calcium is low or normal, while calcimimetics are favored when calcium is elevated or when vitamin D analogs have failed to achieve PTH targets [5]. Triple therapy incorporating all three classes is used in patients with refractory sHPT, though clinical trial data specifically evaluating triple combination regimens remain limited. The KDIGO guidelines endorse combination use but stop short of mandating specific sequencing protocols, leaving clinicians to individualize treatment based on biochemical response, tolerability, and cost [5]. In practice, phosphate control should be addressed first with dietary restriction and binders, followed by targeted PTH suppression using vitamin D analogs or calcimimetics based on serum calcium levels [5].

In real-world clinical settings, treatment decisions are rarely made in isolation. The choice of which agents to combine, in what doses, and in what sequence depends on individual patient factors, including baseline PTH, calcium, phosphate, and alkaline phosphatase levels; dialysis modality and vintage; comorbidities; and tolerability of prior therapies. This complexity is not well captured by clinical trials, which typically evaluate fixed treatment protocols in selected patient populations. Therefore, pragmatic observational studies in routine clinical practice settings are an important complement to controlled trial evidence [3].

Secondary hyperparathyroidism and clinical outcomes

The clinical consequences of uncontrolled sHPT extend well beyond abnormal laboratory values, affecting skeletal integrity, cardiovascular health, and overall survival. At the skeletal level, chronically elevated PTH drives high-turnover renal osteodystrophy, manifesting as osteitis fibrosa cystica, subperiosteal bone resorption, and cortical thinning [8]. These changes substantially increase fracture susceptibility, and dialysis patients experience fracture rates that far exceed those of age-matched individuals without kidney disease [9]. Observational data indicate that among dialysis patients, fractures carry a 3.7-fold increase in subsequent mortality risk, driven by post-fracture immobility, sepsis, and cardiovascular decompensation [9]. Poor PTH control has also been linked to vascular calcification and cardiovascular complications, which remain the leading cause of death in the dialysis population [7,10].

Cardiovascular disease accounts for approximately 40-50% of all-cause mortality in dialysis patients, and disordered mineral metabolism is a major contributing mechanism [7,10]. Hyperphosphatemia and elevated calcium-phosphate product promote vascular smooth muscle cell transdifferentiation into an osteogenic phenotype, resulting in medial arterial calcification, increased arterial stiffness, and left ventricular hypertrophy [10,11]. Large observational data have documented that elevated serum phosphate and calcium-phosphate product are independently associated with cardiac mortality in hemodialysis patients, with risk increasing progressively above normal thresholds [10]. Both very low and very high PTH levels carry independent mortality risk in a U-shaped relationship, with the lowest mortality observed among patients maintaining iPTH within the KDIGO-recommended range of two to nine times the upper limit of normal [7]. Elevated PTH levels have additionally been associated with increased rates of hospitalization and greater healthcare utilization in dialysis populations [6,7]. Some analyses have suggested that calcimimetic therapy may reduce cardiovascular event rates and fracture incidence, though the largest randomized controlled trial failed to confirm a statistically significant mortality benefit [18,19]. These outcome data derive overwhelmingly from Western and Asian trial populations, and whether equivalent associations between PTH control and hard clinical outcomes exist in Middle Eastern dialysis patients, who carry a higher burden of diabetic nephropathy and distinct cardiovascular risk profiles, remains entirely unknown [6].

Research gaps

Despite meaningful advances in sHPT pharmacotherapy, several critical evidence gaps limit the translation of current knowledge into optimal clinical practice, particularly in the GCC and Middle Eastern context. The most fundamental gap concerns clinical trial design: the overwhelming majority of randomized controlled trials have been powered to detect differences in surrogate biochemical endpoints rather than hard clinical outcomes such as fractures, cardiovascular events, hospitalizations, or all-cause mortality. The gap between biochemical efficacy and clinical benefit is starkly illustrated by the largest cinacalcet trial, in which robust and sustained PTH lowering failed to produce a statistically significant reduction in the primary composite cardiovascular endpoint [19]. This disconnect suggests that mineral metabolism is one of the many contributing pathways to cardiovascular death in dialysis patients and that PTH lowering alone is insufficient to meaningfully alter overall risk. Future trials must be designed from the outset with clinically meaningful primary endpoints, adequate follow-up duration, and strategies to minimize treatment discontinuation that complicates intention-to-treat analyses.

The regional evidence gap for Middle Eastern and GCC populations is a distinct and underappreciated problem. Oman and the broader GCC region present an epidemiological profile that differs systematically from Western settings in ways directly relevant to sHPT management: diabetic nephropathy accounts for 46% of incident dialysis cases in Oman [14], a proportion higher than reported in many European dialysis registries; vitamin D deficiency is near-universal due to indoor lifestyles and cultural clothing practices despite high ambient sunlight [12]; dietary phosphate exposure is shaped by regional food patterns; and calcimimetic utilization in GCC dialysis centers is substantially lower than in North American or European centers [6]. No published prospective study has examined sHPT pharmacotherapy outcomes specifically in Omani or GCC dialysis patients. National registry studies, multi-center prospective cohorts, and pragmatic trials within GCC healthcare systems are needed to establish whether existing biochemical targets and treatment algorithms are appropriate for this distinct population.

Combination therapy optimization remains an unresolved practical challenge. Although current guidelines endorse combination pharmacotherapy [5], there is no high-quality evidence guiding the optimal initial agent, the preferred sequence of escalation, or the appropriate dose-adjustment strategy when biochemical targets are not achieved with initial treatment. The real-world complexity of managing sHPT, where calcium, phosphate, PTH, alkaline phosphatase, dialysis adequacy, and drug tolerability must all be balanced simultaneously in individual patients, is poorly captured by clinical trials that test fixed protocols in selected populations. Pragmatic trials and real-world observational studies examining individualized combination approaches in routine dialysis settings are an important unmet need. Phosphate binder non-adherence, with rates of 22% to 74% across dialysis populations [20], represents the single most prevalent modifiable barrier to effective sHPT control and deserves substantially greater attention in both clinical research and quality improvement programs. Finally, the cost-effectiveness of available sHPT therapies has not been evaluated using GCC-specific drug pricing, formulary structures, or healthcare utilization data. Formulary decisions in GCC healthcare systems are currently made without locally relevant economic evidence, creating a risk that access to more effective agents is restricted based on acquisition cost alone.

## Conclusions

sHPT remains one of the most prevalent and clinically consequential complications of ESRD, and the three principal pharmacological classes, i.e., calcimimetics, vitamin D analogs, and phosphate binders, offer a flexible, evidence-based strategy for biochemical control when used individually or in combination, guided by serum calcium, treatment response, and tolerability. However, real-world PTH control remains suboptimal, and no pharmacological intervention has demonstrated a statistically significant mortality benefit in randomized controlled trials, meaning treatment goals should be framed around biochemical control and complication prevention rather than survival claims. Phosphate binder non-adherence remains the single most prevalent modifiable barrier to effective management and warrants explicit clinical attention. A critical and underappreciated evidence gap exists in the Middle Eastern and GCC context, where diabetic nephropathy dominance, endemic vitamin D deficiency, and lower calcimimetic utilization limit the generalizability of existing trial data. Therefore, prospective cohort studies and pragmatic clinical trials in this population are urgently needed to establish whether current biochemical targets and treatment algorithms are appropriate for the patients they are meant to serve.
